# Effect of Cathodal Transcranial Direct Current Stimulation for Lower Limb Subacute Stroke Rehabilitation

**DOI:** 10.1155/2023/1863686

**Published:** 2023-05-27

**Authors:** Qian Duan, Wenying Liu, Jinhui Yang, Ben Huang, Jie Shen

**Affiliations:** ^1^Department of Rehabilitation, The Eighth People's Hospital of Shanghai, Shanghai 200105, China; ^2^Department of Rehabilitation, Shanxi Provincial People's Hospital, Taiyuan 030012, China

## Abstract

**Methods:**

A pilot double-blind and randomized clinical trial. Ninety-one subjects with subacute stroke were treated with cathodal/sham stimulation tDCS based on CGR (physiotherapy 40 min/d and occupational therapy 20 min/d) once daily for 20 consecutive working days. Computer-based stratified randomization (1 : 1) was employed by considering age and sex, with concealed assignments in opaque envelopes to ensure no allocation errors after disclosure at the study's end. Patients were evaluated at T0 before treatment, T1 immediately after the posttreatment assessment, and T2 assessment one month after the end of the treatment. The primary outcome index was assessed: lower limb Fugl-Meyer motor score (FMA-LE); secondary endpoints were other gait assessment and relevant stroke scale assessment.

**Results:**

Patients in the trial group performed significantly better than the control group in all primary outcome indicators assessed posttreatment T1 and at follow-up T2: FMA-LE outcome indicators between the two groups in T1 (*P* = 0.032; effect size 1.00, 95% CI: 0.00 to 2.00) and FMA-LE outcome indicators between the two groups in T2 (*P* = 0.010; effect size 2.00, 95% CI: 1.00 to 3.00).

**Conclusion:**

In the current pilot study, ctDCS plus CGR was an effective treatment modality to improve lower limb motor function with subacute stroke. The effectiveness of cathodal tDCS in poststroke lower limb motor dysfunction is inconclusive. Therefore, a large randomized controlled trial is needed to verify its effectiveness.

## 1. Introduction

Stroke is the third leading cause of disability worldwide, resulting in permanent disability in 15% to 30% of survivors. Gait balance dysfunction is the most common complication in stroke patients, and it is estimated that more than 50% of hemiplegic patients are left with lower limb gait balance dysfunction, which causes falls, increases the risk of trauma, affects independence in daily life, and increases the burden on families and society [1].

Walking is a complicated motor pattern [2]. Dynamic standing balance, strength, motor control, and precise movement of the center of pressure are all necessary for normal walking. Most improvements in standing balance and walking ability after the stroke occur within 5 to 8 weeks [3]. However, rehabilitation training (proprioceptive training, gait walking training, lower limb robotic training, and ankle-foot orthosis) can improve gait balance, but the effect of rehabilitation is relatively slow [4]. Therefore, new rehabilitation techniques have become a hot topic of research. Studies have shown that restoring spontaneous neuroplasticity is crucial in gait recovery [5]. Transcranial direct current stimulation (tDCS) is an efficient, simple, portable, safe, and economical noninvasive brain neuromodulation technique [6] that regulates GABAergic and glutamatergic synapses in the cortex by generating low-intensity direct current (usually 1 to 2 mA) through electrodes placed in the cranial area. Synapses, depending on the polarity of the stimulus, cause a change in the depolarization or hyperpolarization of the resting membrane potential of neurons, increasing or decreasing cortical excitability at the anode and cathode, respectively. In addition, it increases cortical blood flow, promotes cortical reorganization, and noninvasively increases neuroplasticity to improve motor skills [7]. Functional magnetic resonance imaging (fMRI) after anodal tDCS stimulation of the M1 area has been shown to significantly increase functional connectivity in the premotor and motor areas of the stimulated hemisphere and modulates the imbalance of connectivity between the left and right hemispheres [8]. Bicephalic tDCS plus functional electrical stimulation improves reaching motor performance after stroke [9].

Rehabilitation of the lower limbs seriously affects daily life. The study of tDCS to improve gait balance disorders in the lower limbs after stroke is an emerging area of research in which treatment with tDCS promotes neurological remodeling on the affected side of the stroke and a certain degree of improvement in gait balance. Still, the stimulation site is seen mainly in the cranially related area where anodal tDCS is placed [10, 11]. One study by Saeys et al. showed a significant improvement in the Tinetti scores compared to the control group in 31 stroke patients after 16 sessions of 1.5 mA, 20 min anodal tDCS [12]. In a chronic stroke patient, Dumont et al. performed 20 min, 2 mA tDCS combined with motor plank training, with anodal tDCS placed in the motor cortex on the side of the lesion, and found that training reduced anterior-posterior sway of the center of gravity (6.18%), displacement trajectory (3.3%), and sway speed (3.3%) [13].

Until now, there are few reports about treating lower limb gait balance disorder with cathodal transcranial direct current stimulation (ctDCS) after stroke [14], primarily seen in rehabilitating upper limb function. A study reported that ctDCS has no beneficial effects on upper motor deficits and quality of life based after stroke compared to sham tDCS. The Fugl-Meyer assessment of motor recovery- (FMA-) upper limb motor, Barthel index (BI), and stroke impact scale (SIS) were assessed before and after treatment [15]. However, in a study of 59 patients divided into a cathodal stimulation group, virtual reality group, and cathodal stimulation combined with the virtual reality training group, the results showed that ctDCS stimulation combined with the virtual reality training group improved upper limb function more significantly than the other two groups [16]. Andrade et al. showed significant improvements in 6MWT and balance BBS indicators in the lower limb of patients after cathodal tDCS stimulation [17], while Fusco et al. did not find improvements in gait-related indicators in their study [18].

TDCS as a noninvasive tool for stroke therapy has shown great potential in the acute phases after cerebral ischemia [19]. A study [20] explored the effects of ctDCS 6 h after focal forelimb M1 ischemia in mice; ctDCS improved motor functionality of the affected forelimbs without changing the ischemic volume. Motor recovery following an ischemic event is correlated with a decrease in the number of microglial cells in the area surrounding the ischemic core; at the same time, microglia morphology shifted toward a healthier state with less phagocytic anti-inflammatory activity.

The initial acute study conducted to mice treated with cathodal tDCS starting after the first 30 minutes of middle cerebral artery occlusion (MCAO) demonstrated that an even earlier intervention using cathodal tDCS led to positive effects on preserving cortical neurons from the ischemic damage, reducing inflammation, and promoting better clinical recovery compared with sham and anodal treatments [21]. These findings suggest a positive role for early ctDCS in neuroprotective effect and motor recovery. However, the effectiveness of ctDCS in poststroke lower limb function is inconclusive; therefore, a large randomized controlled trial is needed to verify its effectiveness. Our paper is aimed at investigating the clinical rehabilitation efficacy of ctDCS on poststroke lower limb motor dysfunction.

## 2. Methods

### 2.1. Study Design and Settings

This clinical trial is a prospective, single-center, double-blind, randomized clinical trial conducted in the rehabilitation department of our hospital from June 2019 to September 2022. Patients were randomly assigned to receive either 20 days of conventional gait rehabilitation therapy (CGR) combined with ctDCS treatment (trial group) or sham-stimulated tDCS treatment plus CGR (control group). All patients were treated with tDCS for 20 min once a day for 20 consecutive days based on CGR therapy. The primary and secondary outcomes of the patients were evaluated before treatment (T0), immediately after the treatment (T1), and one month after the end of the treatment (T2). All patients were evaluated by the same trained and uninformed rehabilitation physician. All patients provided their informed consent in writing. The study was approved by the Ethics Committee of Shanghai Eighth People's Hospital and is governed according to the principles of the Declaration of Helsinki.

### 2.2. Participants

We prospectively recruited patients with lower limb motor dysfunction after the first stroke diagnosis (including neuroimaging) in the rehabilitation department of our hospital. Anterior circulation stroke can result in highly variable lower limb motor deficits in terms of lesion side, site, type, number, extension, duration, and severity. To address this potential variability, we carefully selected a clinically homogeneous sample for our study. Inclusion criteria are as follows: (1) age ≥ 40 years and ≤80 years; (2) cerebral infarction in the anterior cerebral circulation region with a duration of 2 weeks-8 weeks; (3) able to live independently after stroke onset (modified Rankin score ≤ 3); (4) lower limb muscle strength of the hemiplegic limb ≥ grade IV; (5) informed consent signed by the patient and his family; (6) able to participate in late follow-up assessments; and (7) able to stand for 5 min without assistance, to walk independently for 5 min without gait aids, and cooperate with the completion of the gait assessment. Exclusion criteria are as follows: (1) history of seizures, pacemaker implantation, intracranial metal implants, and increased intracranial pressure; (2) ataxia of the limb, dysarthria, nystagmus, reduced facial expression, signs of upper motor neuron damage, and resting tremor; (3) presence of other neurological disorders in addition to stroke or other conditions that may affect mobility and assessment protocol musculoskeletal problems; (4) individuals with severe combined circulatory or respiratory disease or other malignant lesions of internal organs or pregnancy; and (5) severe cognitive impairment, depression and unable to cooperate with gait assessment.

### 2.3. Randomization and Distribution

Computer-based stratified randomization (1 : 1) was generated, with strata defined by age (40 to 55 years, 56 to 65 years, and 66 to 80 years) and sex. Assignments were concealed in opaque envelopes until the start of the intervention. After disclosure at the end of the study, we ensured that there were no allocation errors.

### 2.4. Evaluation Criteria

The wearable gait analyzer (Beijing Norton-Global Motion Capture Technology) comprises 17 systems across the extremities and trunk. Combined with gait scales, it allows for standardized testing protocols, improved sensitivity, repeatability, accuracy of assessment, and accurate analysis of patient balance and standing function, excluding the influence of patient and assessor personal factors.

The primary outcome was the Fugl-Meyer motor assessment for the lower extremities (FMA-LE), which included supine Achilles and knee tendon reflex activity, lower extremity flexor band movement, lower extremity extensor band movement, seated with band movement, standing separated movement, seated normal reflexes, and supine heel-knee-shin test, to test the motor synergy, reflexes, and coordination ability of the lower limbs, scoring a total of 6 major items and 17 subitems, with scores ranging from 0 to 2 and a maximum score of 34.

Secondary outcome measures were other gait assessment indicators and related stroke scales.

#### 2.4.1. Gait Assessment Scale

*(1) Two-Minute Walking Test (2MWT)*. The patient walks for at least 2 minutes at the usual speed, 10 meters is selected, and the walking time of 10 meters (ten-meter walking test, 10MWT) is recorded by applying a wearable gait analyzer.

*(2) Timed Up and Go Test (TUGT)*. The patient is asked to stand up from the chair, walk 3 meters, turn around, walk back to the chair, and sit down. A wearable forensic step analyzer was used to record the duration.

*(3) Tinetti Performance-Oriented Mobility Assessment (Tinetti POMA)*. There are 10 items out of 16 for the balance section and eight items out of 12 for the gait section.

#### 2.4.2. Related Stroke Scale

The instrumental daily living ability score (IADL) assesses the ability of patients to maintain an independent life and Hamilton depression scale (HAMD).

### 2.5. Treatment Procedures

All subjects were treated with cathodal/sham tDCS stimulation based on CGR (physiotherapy 40 min/d and occupational therapy 20 min/d). The stimulation site of tDCS was the location of the projection of the M1 area of the primary motor cortex of the lower limb of the healthy hemisphere on the body surface (according to the 10-20 International EEG System body surface localization method, the M1 area of the brain corresponds to C3/C4). The anode was placed in the contralateral supraorbital area. All subjects received two sessions (20 min once a day for 20 consecutive days) of tDCS treatment. The applied transcranial direct current stimulator was an IS200 intelligent stimulator (Sichuan Intelligent Electronic Industrial Company), and the stimulating electrodes were 5 cm × 5 cm isotonic saline gelatin sponge electrodes. The electric current smoothly increased to 2 mA and fell slowly for more than 40 seconds to ensure that the subjects tolerated the tingling sensation. The control group then received stimulation of 2.0 mA for 40 s (including 20 s each for the rise and fall of the current) at the beginning and ending time, causing a local skin itch similar to that of the test group. The treatment was completed by the same professional rehabilitation therapist using the same tDCS machine. Subjects were required to receive at least 80% of the treatments during the treatment period, i.e., to complete at least 16 treatments, or they were considered to be dislodged.

### 2.6. Statistical Analysis

During the trial, the evaluator was blinded to the trial group. The exploratory data analysis and the Shapiro-Wilk test were performed to determine the normality of the data distribution. Continuous variables were analyzed using *t*-tests or Mann-Whitney *U* tests, and the results were expressed as means ± standard deviations (SD) or medians of interquartile ranges (IQRs). The difference between the two means and the 95% confidence interval (CI) for the difference was calculated. The difference between the two medians and the 95% CI for the difference was calculated with the Hodges-Lehmann estimate. The Bonferroni correction was used when investigating multiple within-group comparisons, resulting in *P* < 0.016 as the significance threshold (Benjamini et al., 1995). For categorical variables, counts and percentages are shown. Comparisons between groups at baseline for categorical variables were tested with the *χ*^2^ test. The significance level was set at a two-sided *P* value of less than 0.05. All analyses were performed using SPSS version 20.0 (SPSS Inc.) and GraphPad Prism 7 for Windows software (GraphPad Software Inc., La Jolla, CA, USA).

## 3. Results

**Figure 1:**
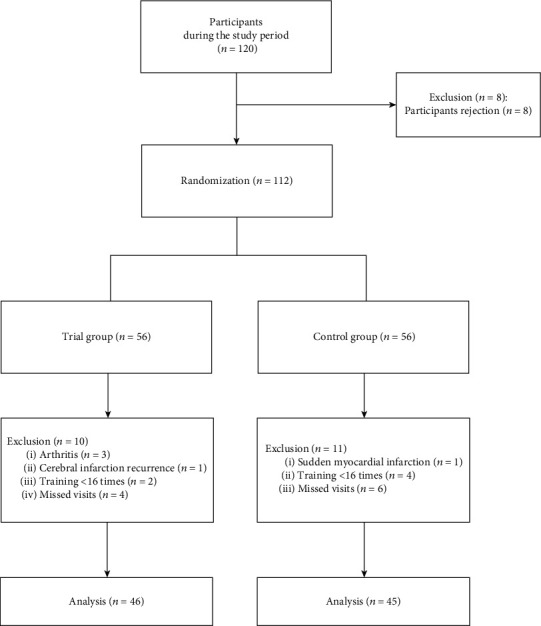
Flow diagram showing the number of records identified and the number of included/excluded studies according to the described evaluation criteria.

Out of the 120 patients who met the inclusion criteria, 112 agreed to participate. Nineteen patients had terminated the study early. Therefore, 91 patients completed the follow-up assessment and were included in the analysis (Figure 1). Among them, 41 (45.1%) were male, with a mean age (SD) of 66.20 (9.51) years. There were no statistically significant differences in the demographic and clinical characteristics of the patients at the time of the T0 evaluation, as detailed in Table 1 (*P* > 0.05).

### 3.1. The Primary Outcome

**Figure 2:**
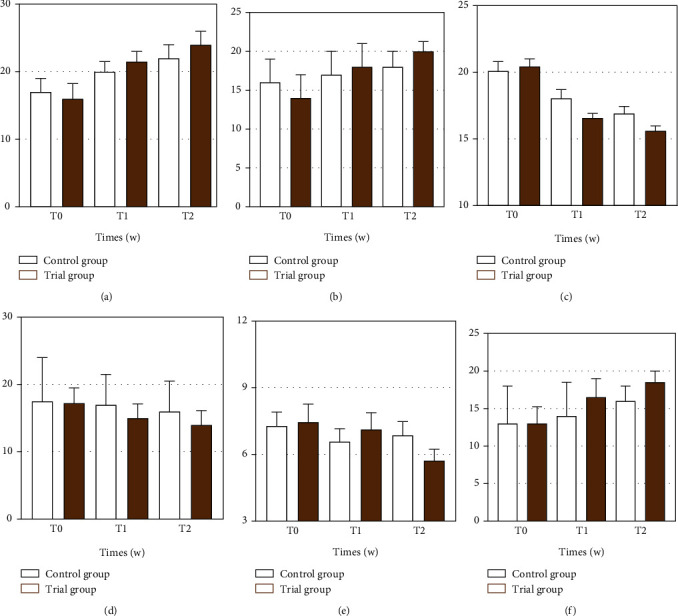
Changes over time in lower limb function: (a) Fugl-Meyer motor score of the lower extremity; (b) Tinetti Gait Balance Scale; (c) timed up and go test; (d) ten-meter walking test and stroke scale assessment tests; (e) Hamilton depression scale; and (f) instrument ability to perform daily living score. Notes: these are in comparison with the baseline level. The black line (a, b, d, f) represents the third quartile. The black line (c, e) represents the standard error of the mean. T0: pretreatment assessment; T1: posttreatment immediately assessment; T2: assessment one month after the end of the treatment.

As shown in Table 2 and Figure 2, a comparison between groups showed that patients in the trial group performed significantly better than the control group in all primary outcome indicators evaluated at T1 and T2. There was no significant difference in the FMA-LE score (Figure 2(a)) (*P* = 0.398) between the two groups at T0. The FMA-LE outcome indicators were better in the trial group than in the control group at T1 (*P* = 0.032; effect size 1.00, 95% CI: 0.00 to 2.00); the FMA-LE outcome was better in the trial group than in the control group (*P* = 0.010, effect size 2.00; 95% CI: 1.00 to 3.00) at T2. The comparisons within groups are shown in Table 2 (Bonferroni correction applied).

### 3.2. Secondary Results

As shown in Table 2, no statistically significant differences were observed between the groups before treatment T0: the Tinetti score (*P* = 0.123), the 10MWT score (*P* = 0.257), and the TUGT score (*P* = 0.714) were not statistically different.

For all secondary outcome measures of motor function of the limb assessed at T1, comparisons between groups showed that the trial group had better results than the control group, with significant improvements compared to the control group. Among all secondary outcome measures of motor function of the lower limbs in T2, the trial group outperformed the control group except for the outcome measure 10 MWT, which was significantly improved compared to the control group (Figure 2(d)). The 10MWT outcome indicator was better in the trial group than in the control group between-group comparisons at T1 (*P* = 0.007; effect size -2.00, 95% CI: -3.50 to -0.50), and there were no between-group differences at T2 (*P* = 0.099l; effect size -1.00, 95% CI: -3.00 to -0.00).

The Tinetti score outcome indicator (Figure 2(b)) was better in the trial group than in the control group at T1 (*P* = 0.031; effect size 2.00; 95% CI: 0.00 to 3.00) and T2 (*P* = 0.048; effect size 1.00; 95% CI: 0.00 to 3.00); the TUGT score outcome indicator of the trial group (Figure 2(c)) was better than the control group between the two groups at T1 (*P* = 0.049; effect size -1.48; 95% CI: -2.93 to -0.02) and T2 (*P* = 0.043; effect size -1.30; 95% CI: -2.56 to -0.04); in addition, no significant differences were found between the two groups of subjects evaluated at different periods on the mood-related scale HAMD (Figure 2(e)), with no significant improvement overall; there were no significant differences in HAMD score indicators between groups at T1 (*P* = 0.561; effect size 0.55; 95% CI: -1.33 to 2.43), and no significant difference between groups was found at T2 (*P* = 0.156, the effect size was -1.15; 95% CI: -2.74 to 0.45).

Comparisons between groups showed that the outcome indicators of the IADL (Figure 2(f)) were better in the trial group than in the control group at different periods, with the IADL score between the two groups at T1 (*P* = 0.036; effect size 2.00; 95% CI: 0.00 to 3.00) and T2 (*P* = 0.028; effect size 2.00; 95% CI: 0.00 to 3.00). Within-group data and the results of intragroup comparisons are shown in Table 2 and Figure 2. Group data and within-group comparisons are reported in Table 2 and Figure 2.

## 4. Discussion

In this study, ctDCS combined with CGR (trial group) was compared with sham-tDCS combined with CGR (control group). The wearable gait analyzer was used to standardize the analysis of patients' lower limb gait function. The results showed that the trial group performed significantly better than the control group in lower limb motor function (FMA-LE) at posttreatment T1 and follow-up assessment T2. This suggests that ctDCS plus CGR is an effective method to improve the motor synergy, reflex, and coordination function of the lower limbs in patients with subacute stroke. Furthermore, in terms of the secondary outcomes, the effects of the trial group were superior in the control group except for the 10MWT outcome at T2, and the patients improved balance, increased walking ability, and reduced the risk of falls.

The mechanism of the efficacy of tDCS in lower limb dysfunction after stroke may explain our results to some extent. The current mechanism is still unclear, and the main consideration is based on the following mechanisms: the interhemispheric inhibition (IHI) theory [22]. In healthy individuals, the excitability of cortical motor neurons is balanced between the two hemispheres, but after stroke, the excitability of the focal hemisphere is reduced while the excitability of the healthy hemisphere is compensated by an increase in excitability, resulting in an imbalance between the hemispheres. TDCS stimulation can reestablish the balanced relationship between the bilateral hemispheres and improve motor function by exciting the affected side or inhibiting the activity of the healthy hemisphere. Modulation of synaptic plasticity: modulation of the synaptic microenvironment, activation of brain-derived nerve growth factor, and tyrosine receptor kinase B [23]. It induces the expression of N-methyl-D-aspartate (NMDA) receptors and inhibits the activity of *γ*-aminobutyric acid (GABA), which induces neuroplasticity and improves motor learning skills while producing long-lasting inhibition or enhancement, resulting in after-effects. Application of M1 zone anodic tDCS (2 mA, 10 min) in healthy adults is effective in reducing the response time to ankle selection compared to sham stimulation treatment [24]. Anodal-tDCS in the M1 area (10 times, 20 min) significantly improved lateral gastrocnemius muscle spasm and increased anterior tibialis, muscle activity, and balance in stroke patients [25]; tDCS may prove to be a promising intervention to improve functional recovery of the lower extremity. Metaplasticity [26]: metaplastic changes, which modulate neural plasticity by adjusting the balance between synaptic input and neuronal firing, promote homeostatic synaptic plasticity and associate plasticity. Any recent neural synaptic activity can affect ongoing activity, and preconditioning neural networks may induce synaptic homeostatic changes. This is related to compensatory upregulation at postsynaptic membrane receptors due to inhibition, resulting in the “rebound effect,” where neurons become more excitable due to initial downregulation induced by cathodal tDCS, which can be reversed by conditioning cathodal tDCS. Additionally, tDCS may induce changes in neurotrophin concentration, gene expression, and modulation of glial cell function, further contributing to its effects on metaplasticity [27].

The ctDCS reduces the excitability of the cerebral cortex, downregulating the contralateral inhibitory pathway on inhibition of the ipsilateral hemisphere, increasing the interhemispheric connectivity of specific brain regions to play a crucial role, and thus improving motor function of the lower limb [28]. Some researchers reported that cathodal tDCS stimulation of the healthy hemisphere in epileptic patients may inhibit hyperexcitability of the healthy hemisphere [29]. However, one study did not reveal significant improvement in upper limb function and hand dexterity in patients treated with ctDCS in the early stages of stroke [18]. In our trial, ctDCS stimulation during the subacute stroke rehabilitation phase confirmed the improvement of lower limb motor and balance functions and reduced the risk of falls.

Moreover, as lower limb motor function improved, the outcome of IADL was better in the trial group than in the control group, improving the patient's life independence and the possibility of returning to work and society. However, improved lower limb motor function was not associated with decreased HAMD levels, improved pessimism, and increased motivation, while recent studies have shown that recovery of stroke function is positively associated with mood [30].

This is a limited pilot study. To some certain extent, it provides reference and guidance for rehabilitating patients with subacute stroke lower limb disorders in clinical practice. Still, it should be emphasized that the strength of our conclusions is limited. First of all, the sample size was small. The number of enrolled patients may have hindered assessing the effect of cathodal tDCS treatment, so our study can be considered a pilot. To further confirm our findings, randomized controlled trials involving larger subject populations are needed. Secondly, tDCS has no optimal parameters at present, including electrode placement, intensity, and timing relative to rehabilitation interventions, as well as patient characteristics which are limited in the previous studies [31, 32]. A meta-analysis of 8 studies that administered ≥5 sessions of tDCS therapy and used the Fugl-Meyer upper extremity scale (FM-UE) as an outcome measure shows that there was a better motor recovery in the trial group compared to the sham group, and a positive dose-response relationship was found with current density (*P* = 0.017) and charge density (*P* = 0.004), but not with current amplitude [33]. In contrast to 1-1.5 mA tDCS stimulation in the upper limb cortex, a previous study showed that 2 mA stimulation was superior in the lower limb cortex, which is located in the interhemispheric fissure and is deeper in the cerebral cortex [34]. The 2 mA anodal tDCS stimulation of the affected M1 region significantly enhanced force production in knee extensors in patients with subacute stroke [35] and postural stability [36]; thus in our study, we applied 20-minute and 2 mA tDCS treatment parameters. The optimal parameters should be explored in future studies. Third, we did not perform any neurophysiological assessment and imaging evaluation, such as motor-evoked potential MEP recordings, which could provide a measure of cortical motor excitability; functional neurological changes were analyzed by functional magnetic resonance. Fourth, the present study lacks a comparison with anodal tDCS and other brain stimulation techniques (TMS) [37] which can be a helpful noninvasive tool to evaluate cortical excitability, neural plasticity, and underlying transmission pathways. TMS studies have shown enhanced cortical excitability and plasticity in VCI patients, which may be considered as an adaptive response to disease progression [38].

## 5. Conclusion

In conclusion, our study reveals that conventional gait rehabilitation combined with ctDCS is an effective treatment for improving gait balance disorders in the lower limb of patients with subacute stroke. In future studies study, the sample size should be further expanded. At the same time, the study would combine wearable gait analyzer or functional MRI imaging changes, MEP, to evaluate the outcome. Furthermore, exploring different diversified sites for tDCS with different parameters is essential. Accordingly, determining the optimal parameters for the relevant sites will help improve the rigorousness of the treatment effect. The ctDCS therapy will truly become a new clinical technique for treating patients with lower limb movement disorders after stroke, improving patients' quality of life and accelerating the return of patients to social life.

## Figures and Tables

**Table 1:** Baseline demographic and clinical characteristics.

Characteristic	Mean (SD)/median (IQR)		*P* value
	Trial group (*n* = 46)	Control group (*n* = 45)	
Age (y)	65.83 (8.8)	66.58 (10.3)	0.708
Male (no., %)	24 (52.2)	17 (37.8)	0.168
Weight (kg)	65 (60.0, 75.0)	64 (56.5, 70.0)	0.233
Medical history (no., %)			
Currently smoking	15 (32.6)	20 (44.4)	0.246
Diabetes	23 (50.0)	26 (57.8)	0.457
Myocardial infarction	12 (26.1)	9 (20.0)	0.491
Atrial fibrillation	17 (37.0)	16 (35.6)	0.889
Hypertension	40 (87.0)	35 (77.8)	0.250
Medication (no., %)			
Antiplatelet drug	43 (93.5)	43 (95.6)	0.664
Antihypertensive drugs	34 (73.9)	29 (64.4)	0.328
Hypoglycemic drug (hypoglycemic)	20 (43.5)	28 (62.2)	0.073
Lipid-lowering drugs (lipid-lowering)	16 (35.6)	11 (24.4)	0.250
Months poststroke (w)	5.0 (3.0, 7.0)	4.0 (2.0, 6.0)	0.071
NIHSS (mean ± SD)	6.41 (3.4)	6.84 (4.2)	0.594

Abbreviations: NIHSS: National Institutes of Health Stroke Scale. Two groups shared the same overall characteristics as listed without significant differences between the groups (*P* > 0.05).

**Table 2:** The results of lower limb function (FMA-LE, 10MWT, TUGT, and Tinetti) and stroke scale assessment tests (HAMD and IADL).

	Group	T0	T1	Effect size (95% CI)	T2	Effect size (95% CI)	Within-group comparisons		
							T1 vs. T0	T2 vs. T0	T1 vs. T2
							*P* value (*Z*)	*P* value (*Z*)	*P* value (*Z*)
FMA-LE	Trial	16.0 (14.00, 18.25)	21.50 (19.00, 23.00)	1.00 (0.00 to2.00)	24.00 (22.00, 26.00)	2.00 (1.00 to3.00)	*P* < 0.001 (4.565)^∗^	*P* < 0.001 (7.087)^∗^	*P* < 0.001 (2.522)^∗^
Median (IQR)	Control	17.0 (15.50, 19.00)	20.00 (18.00, 21.50)		22.00 (20.00, 24.00)		*P* < 0.001 (3.088)	*P* < 0.001 (5.444)^∗^	*P* < 0.001 (2.355)^∗^
10MWT (s)	Trial	17.25 (14.00, 19.50)	15.00 (13.50, 17.13)	-2.00 (-3.50 to -0.50)	14.00 (12.00, 16.13)	-1.00 (-3.00 to -0.00)	0.154 (1.626)	0.003 (2.800)^∗^	0.473 (1.173)
Median (IQR)	Control	17.5 (14.50, 24.00)	17.00 (15.00, 21.50)		16.0 (12.75, 20.50)		0.941 (1.068)	0.025 (2.824)	0.297 (1.755)
TUGT (s)	Trial	20.44 (3.82)	16.58 (2.35)	-1.48 (-2.93 to -0.02)	15.62 (2.42)	-1.30 (-2.56 to -0.04)	*P* < 0.001 (3.836)^∗^	*P* < 0.001 (4.820)^∗^	0.163 (0.957)
Mean (SD)	Control	20.11 (4.66)	18.06 (4.37)		16.92 (3.53)		0.067 (2.056)	0.001 (3.189)^∗^	0.613 (1.133)
Tinetti	Trial	14.0 (12.00, 17.00)	18.00 (16.00,21.00)	2.00 (0.00 to 3.00)	20.00 (18.00, 21.50)	1.00 (0.00 to 3.00)	*P* < 0.001 (4.261)^∗^	*P* < 0.001 (5.435)^∗^	0.250 (1.174)
Median (IQR)	Control	16.0 (13.00, 19.00)	17.00 (15.00, 20.00)		18.00 (16.00, 20.00)		0.120 (1.151)	*P* < 0.001 (2.844)^∗^	0.209 (1.333)
IADL	Trial	13.0 (12.00, 15.25)	16.50 (15.00, 19.00)	2.00 (0.00 to 3.00)	18.50 (16.00, 20.00)	2.00 (0.00 to 3.00)	*P* < 0.001 (2.913)^∗^	*P* < 0.001 (4.130)^∗^	0.313 (1.217)
Median (IQR)	Control	13.0 (11.00, 18.00)	14.00 (12.00, 18.50)		16.00 (14.00, 18.00)		0.595 (1.089)	0.024 (2.267)	0.493 (1.178)
HAMD	Trial	7.46 (5.48)	7.13 (5.05)	0.55 (-1.33 to 2.43)	5.72 (3.54)	-1.15 (-2.74 to 0.45)	1.000 (0.326)	0.247 (1.739)	0.472 (1.413)
Mean (SD)	Control	7.29 (4.15)	6.58 (3.91)		6.87 (4.11)		1.000 (0.711)	1.000 (0.422)	1.000 (0.289)

Abbreviations: FMA-LE: Fugl-Meyer motor score of the lower extremity; 10MWT: 10-minute walk test; TUGT: timed up and walk test; Tinetti: Tinetti gait balance scale; IADL: instrumental ability to perform daily living score; HAMD: Hamilton depression scale; IQR: interquartile range; SD: standard deviation; CI: confidence interval. ^∗^Statistically significant after Bonferroni's correction (*P* < 0.016).

## Data Availability

The data that support the findings of this study are available from the corresponding author upon reasonable request.
